# *OsExo70B1* Positively Regulates Disease Resistance to *Magnaporthe oryzae* in Rice

**DOI:** 10.3390/ijms21197049

**Published:** 2020-09-25

**Authors:** Hongna Hou, Jianbo Fang, Jiahui Liang, Zhijuan Diao, Wei Wang, Dewei Yang, Shengping Li, Dingzhong Tang

**Affiliations:** 1 State Key Laboratory of Ecological Control of Fujian-Taiwan Crop Pests, Key Laboratory of Ministry of Education for Genetics, Breeding and Multiple Utilization of Crops, Plant Immunity Center, Fujian Agriculture and Forestry University, Fuzhou 350002, China; Hongna_0506@163.com (H.H.); jianbofang@163.com (J.F.); 13437898434@163.com (J.L.); diaojuan1981@126.com (Z.D.); vic_0214@163.com (W.W.); dewei-y@163.com (D.Y.); 2 College of Life Science, Fujian Agriculture and Forestry University, Fuzhou 350002, China; 3 Rice Research Institute, Fujian Academy of Agricultural Sciences, Fuzhou 350019, China

**Keywords:** rice, *OsExo70B1*, plant disease resistance, OsCERK1, rice blast disease, plant immunity

## Abstract

The exocyst, an evolutionarily conserved octameric protein complex, mediates tethering of vesicles to the plasma membrane in the early stage of exocytosis. *Arabidopsis* Exo70, a subunit of the exocyst complex, has been found to be involved in plant immunity. Here, we characterize the function of *OsExo70B1* in rice. *OsExo70B1* mainly expresses in leaf and shoot and its expression is induced by pathogen-associated molecular patterns (PAMPs) and rice blast fungus *Magnaporthe oryzae* (*M. oryzae*). Knocking out *OsExo70B1* results in significantly decreased resistance and defense responses to *M. oryzae* compared to the wild type, including more disease lesions and enhanced fungal growth, downregulated expression of pathogenesis-related (PR) genes, and decreased reactive oxygen species accumulation. In contrast, the *exo70B1* mutant does not show any defects in growth and development. Furthermore, *OsExo70B1* can interact with the receptor-like kinase OsCERK1, an essential component for chitin reception in rice. Taken together, our data demonstrate that *OsExo70B1* functions as an important regulator in rice immunity.

## 1. Introduction

Plants have evolved two layers of the innate immunity system, pattern-triggered immunity (PTI) and effector-triggered immunity (ETI), to defend against pathogens [1,2,3,4]. In PTI and ETI, many defense-related proteins must be transported to the suitable sites and then exercise their function [5,6], which is partly dependent on the secretory trafficking system. In plants, the defense-related secretory trafficking is associated with three types of membrane vesicles: the trans-Golgi network/early endosome (TGN/EE), the multivesicular body (MVB), and the exocyst positive organelle (EXPO) [6].

EXPO is uniquely labeled by components of the exocyst complex, an evolutionarily conserved octameric tethering factor, which mediates post Golgi vesicle fusion with the plasma membrane (PM) and plays a major role in exocytosis [7,8,9,10,11]. The exocyst complex consists of eight subunits: Sec3, Sec5, Sec6, Sec8, Sec10, Sec15, Exo70 and Exo84 [12]. Among them, Exo70 is a key member of the exocyst complex and has been found to be widely present in yeast, mammals and plants [13,14]. In yeast and mammals, there is one Exo70 gene, while there are multiple copies of Exo70 genes in plants [15] ranging from 21 to 47 Exo70 members in potatoes, Arabidopsis, Populus trichocarpa, wheat and rice [16,17,18,19,20]. Exo70 genes have been duplicated independently in the moss, lycophyte and angiosperm lineages, and in the subsequent lineage-specific multiplications which are represented by nine subgroups (Exo70A–Exo70I) [15] and their function ranges from growth, development to biotic and abiotic stresses [21,22,23,24,25,26,27,28].

In recent years, increased evidence indicates that some exocyst complex members, especially Exo70 family genes, are involved in plant immunity. For instance, in Arabidopsis, Exo70B2 and Exo70H1 are upregulated after treatment with the elicitor elf18, and *exo70B2* and *exo70H1* mutants are more susceptible to Pseudomonas syringae pv. maculicola compared with the wild type [29]. PUB22-mediated ubiquitination and degradation of Exo70B2 contribute to PAMP-triggered responses [21]. Exo70B1 is implicated in autophagy-related transport to the vacuole. Loss of function of Exo70B1 causes reduction in internalized autophagic bodies inside the vacuole [30], along with ectopic hypersensitive responses [23,30] and enhanced resistance to several pathogens, including the powdery mildew Golovinomyces cichoracearum, the bacterial pathogen *Pseudomonas syringae* pv. *tomato* (*Pto*) DC3000, and the oomycete pathogen Hyaloperonospora arabidopsidis Noco2 [23]. Recently, it has been found that Exo70B1-enhanced disease resistance and cell death in the *exo70B1* mutant are dependent on TIR-NBS2 (TN2) and calcium-dependent protein kinase 5 (CPK5) [23,31]. Moreover, OsSEC3A, an important subunit of the exocyst complex in rice, participate in rice immunity by interacting with rice SNAP025-type SNARE protein OsSNAP32 and phosphatidylinositol-3-phosphate (PI(3)P) [32]. Further studies showed that some pathogen effectors can exploit exocyst subunits as host targets to modulate defense. For instance, Phytophthora infestans RXLR Effector AVR1 binds SEC5 to disturb vesicle trafficking thereby suppressing host defense [33]. The effector AvrPtoB, an E3 ligase from *Pto* DC3000, ubiquitinates Exo70B1 and mediates Exo70B1 degradation [34]. In addition, the effector AVR-Pii forms a complex with two rice Exo70 proteins, OsExo70F2 and OsExo70F3. Remarkably, OsExo70F3 was found to be necessary for Pii-dependent resistance [35]. Increased evidence shows that exocyst complexes play important roles in plant immunity, but the functions and regulation of these family genes are still largely unknown.

In rice, there are three Exo70B members, *OsExo70B1*, *OsExo70B2* and *OsExo70B3* [17], but their functions are still unknown. Here, we characterize the function of *OsExo70B1* in rice. *OsExo70B1* expression level is induced by pathogen-associated molecular patterns (PAMPs) and rice blast fungus *M. oryzae*. Then, we generated rice *exo70B1* mutants by CRISPER/Cas9 method and we found that the *exo70B1-1* and *exo70B1-2* mutants both display enhanced susceptibility to *M. oryzae*. Further study indicated that *OsExo70B1* interacts with OsCERK1, which is essential for chitin-induced defenses in plants [36,37,38]. Our results indicated that *OsExo70B1* positively regulates disease resistance in rice.

## 2. Results

### 2.1. Characterization of OsExo70B1

**Figure 1:**
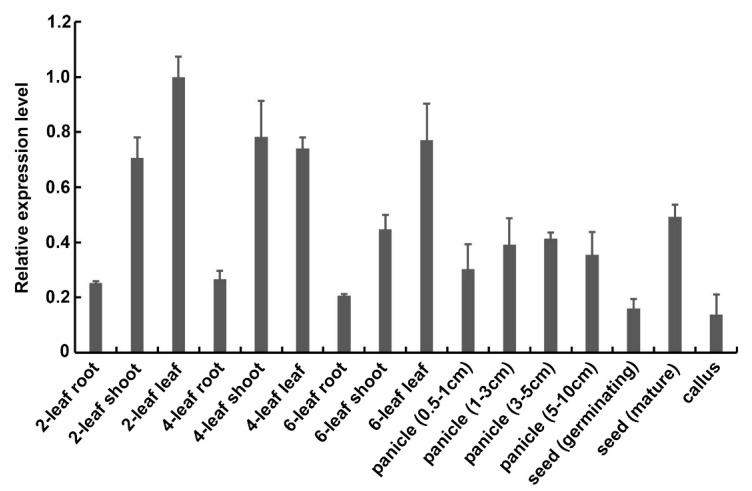
Expression patterns of *OsExo70B1*.

Previous studies indicated that the exocyst subunit Exo70B1 is involved in the immune response to different pathogens in *Arabidopsis thaliana* [23,31]. However, the function of Exo70B1 in rice is still unknown. In order to understand its function, we first examined the expression patterns of *OsExo70B1* in different developmental stages using quantitative reverse transcription-PCR (qRT-PCR). As shown in Figure 1, *OsExo70B1* expressed in all tissues tested here, including root, shoot, leaf, panicle, seed and callus, but it predominantly expressed in the leaf and shoot, then in panicle and seed.

**Figure 2:**
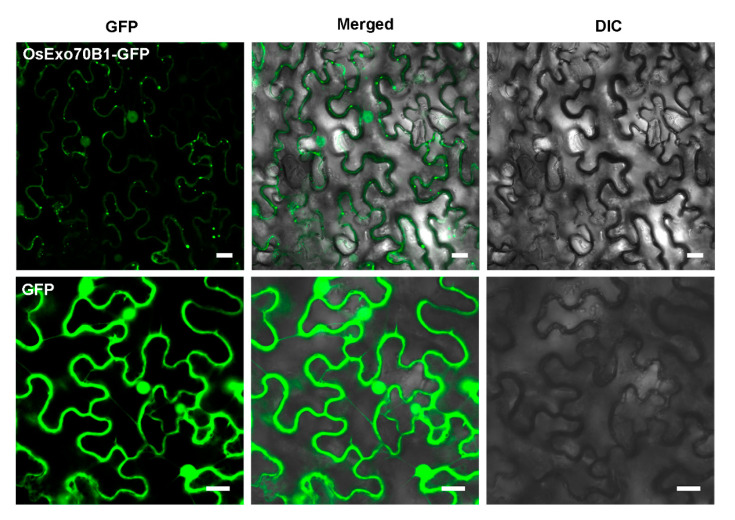
Subcellular localizations of *OsExo70B1*.

Then, in order to examine the subcellular localization of *OsExo70B1*, the *OsExo70B1* fused with the green fluorescent protein (GFP) was expressed under the control of the 35S promoter in *N. benthamiana* leaves by agrobacterium-mediated genetic transformation. We observed clear fluorescence signals in the cytoplasm, nucleus, and plasma membrane (PM) (Figure 2), indicating that *OsExo70B1* distributed throughout the whole cell, which is similar to some Exo70 members in *Arabidopsis* and *H. villosa*, such as AtExo70B1, Exo70D1-V and Exo70F2-V [20,39]. Interestingly, *OsExo70B1* formed some strong dotted fluorescence in the cytoplasm and plasma membrane, which is similar to Exo70A1, Exo70B1 and Exo70E2 in *Arabidopsis* [24,30,40].

Determination of the expression patterns of *OsExo70B1* via qRT-PCR. RNA samples were extracted from different tissues of ZH11, including roots, shoots and leaves of three different developmental stages, panicles of different length, germinating and mature seeds and callus. Data represent the mean and standard deviation of three biological replicates. Three technical replicates for each biological sample were used. The asterisk indicates significant differences (* *p* < 0.05; Student’s *t*-test).

Confocal microscopy observation of *OsExo70B1*-GFP in *N. benthamiana* leaves. The results showed that *OsExo70B1*-GFP was expressed in the nucleus, cytoplasm and plasma membrane. Bar = 20 μm.

### 2.2. Transcription of OsExo70B1 Is Induced by PAMPs and *M. oryza*

**Figure 3:**
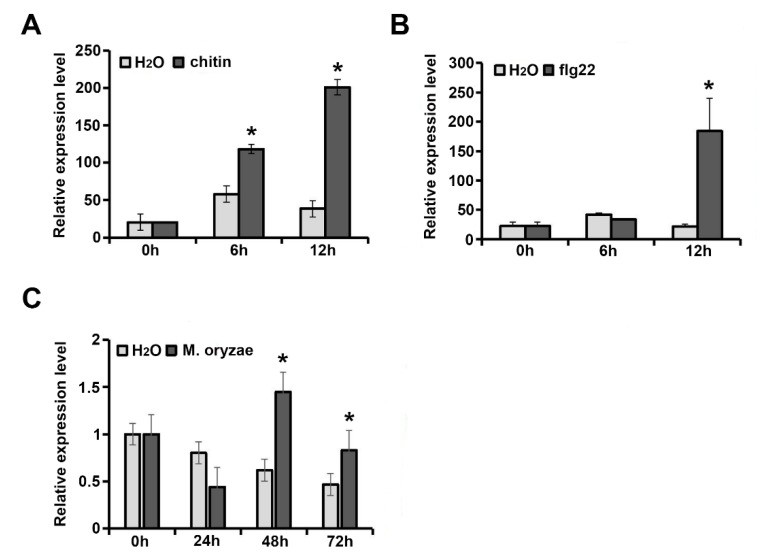
Transcripts of *OsExo70B1* are induced by pathogen-associated molecular patterns (PAMPs) and *M. oryzae*. (**A**) Expression analysis of *OsExo70B1* after chitin treatment, with H_2_O set as a control. (**B**) Expression analysis of *OsExo70B1* after flg22 treatment, with H_2_O set as a control. (**C**) Expression analysis of *OsExo70B1* after *M. oryzae* inoculation, with H_2_O set as a control. Data represent the mean and standard deviation of three biological replicates. Three technical replicates for each biological sample were used. The asterisk indicates significant differences (* *p* < 0.05; Student’s *t*-test).

It has been reported that AtExo70B1 regulates immunity in *Arabidopsis* [21,34,41]. Pathogen-derived elicitors, often called pathogen-associated molecular patterns (PAMPs), are recognized by pattern-recognition receptors (PRRs) of hosts, and activate plant immune responses [1,2,3,4]. To test whether Exo70B1 is involved in the immune response regulation in rice, the expression of *OsExo70B1* upon PAMPs treatment was analyzed. When the wild-type ZH11 seedings grew to four weeks, leaf segments that were 2–3 cm in length were collected and treated with flg22 or chitin, two well-characterized PAMPs. Then, the expression levels of *OsExo70B1* at different time periods were detected. The results showed that the expression level of *OsExo70B1* was significantly increased after flg22 and chitin treatments (Figure 3A,B). Furthermore, we also detected the transcription levels of *OsExo70B1* after *M. oryzae* inoculation and found that *OsExo70B1* was also upregulated upon *M. oryzae* infection (Figure 3C). The results above indicated that the expression of *OsExo70B1* is induced by PAMPs and *M. oryzae*, revealed its potential roles in rice immunity regulation.

### 2.3. Knocking out OsExo70B1 Compromises Plant Resistance to M. oryzae

**Figure 4:**
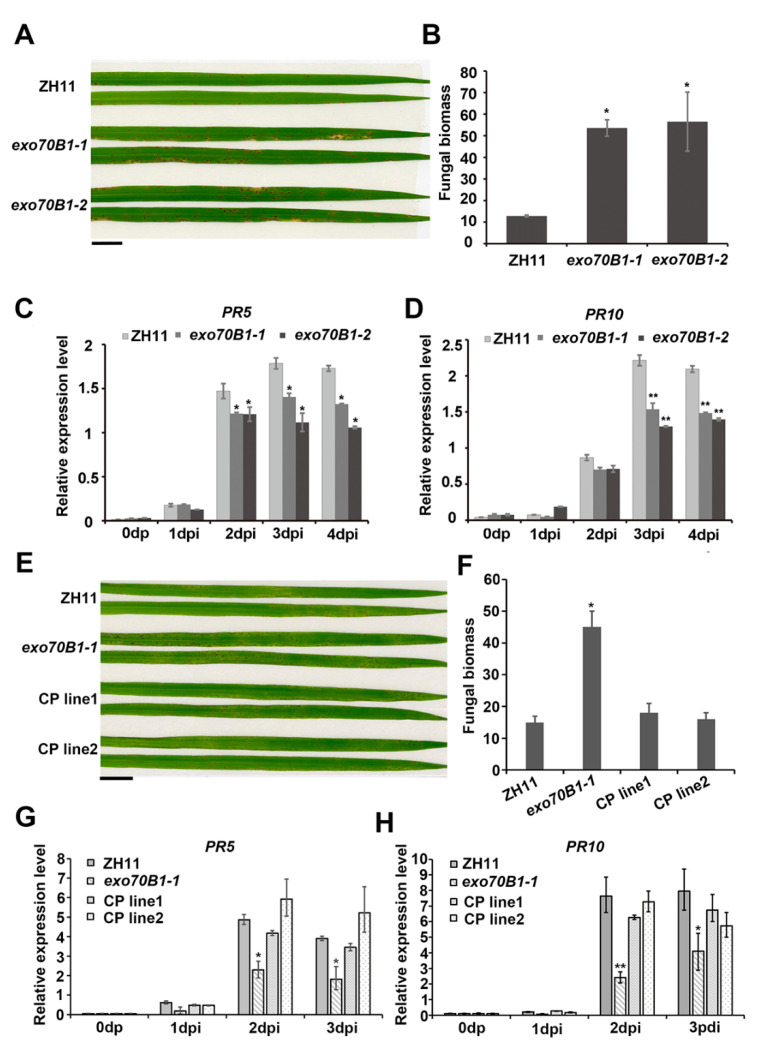
Knocking out Exo70B1 decreased plant resistance to *M. oryzae*. (**A**) *exo70b1-1* and *exo70B1-2* produced more diseased lesions compared to the wild type after inoculation with Guy11 using the spraying method. Bar = 1 cm. (**B**) Relative fungal amounts as determined by qPCR of the *M. oryzae Pot2* gene compared to the *Ubiquitin* gene and compared to ZH11. Data represent the mean and standard deviation of three biological replicates. Three technical replicates for each biological sample were used. Asterisks represent significant differences (* *p* < 0.05, Student’s *t*-test). (**C**,**D**) Transcripts of Os*PR5* and Os*PR10* were both significantly downregulated in *exo70b1-1* and *exo70B1-2* compared to the wild type. Data represent the mean and standard deviation of three biological replicates. Three technical replicates for each biological sample were used. Asterisks represent significant differences relative to wild-type ZH11 plants (* *p* < 0.05, ** *p* < 0.01, Student’s *t*-test). (**E**) Rice disease lesions caused by *M. oryzae* locally inoculated (10^5^ spores/mL) on leaves of ZH11, *exo70B1-1* and *exo70B1-1* complementary plants (CP). Bar = 1 cm. (**F**) Relative fungal amounts of ZH11, *exo70B1-1* and *exo70B1-1* complementary plants (CP). Data represent the mean and standard deviation of three biological replicates. Three technical replicates for each biological sample were used. Asterisks represent significant differences (* *p* < 0.05, Student’s *t*-test). (**G**,**H**) Transcripts of Os*PR5* and Os*PR10* were examined in the wild type, *exo70B1-1* and the *exo70B1-1* complementary plants (CP). Data represent the mean and standard deviation of three biological replicates. Three technical replicates for each biological sample were used. Asterisks represent significant differences relative to wild-type ZH11 plants (* *p* < 0.05, Student’s *t*-test).

To further investigate the genetic role of *OsExo70B1* in rice immunity, we generated the *exo70B1* mutant by CRISPR-Cas9 methods in the ZH11 background. As shown in Figure S1, we obtained two *OsExo70B1* allelic mutants, designated *exo70B1-1*, which has a T base insertion at the 245 position that resulted in a code-shift mutation from Pro_84,_ and *exo70B1-2*, which has a C base insertion at the 145 position that resulted in a code-shift mutation from His_49,_ which are likely to be two loss-of-function mutants. Moreover, we also examined the transcripts of *OsExo70B1* in both *exo70B1-1* and *exo70B1-2* mutants. The expression level of *OsExo70B1* was not changed in the exo70B1 mutants compared to the wild type (Figure S2). We then inoculated *exo70B1-1*, *exo70B1-2* and the wild-type plants with the *M. oryzae* isolate Guy11. Three days post-inoculation, the *exo70B1-1* and *exo70B1-2* mutants displayed enhanced susceptibility and showed more and extended lesions, while the wild-type had only a few lesions (Figure 4A). Consistent with this, the fungal biomass was accumulated at a higher level in the infected *exo70B1-1* and *exo70B1-2* leaves than in the wild type.

Moreover, we measured the transcription levels of pathogenesis-related genes using qRT-PCR. *OsPR5* and *OsPR10*, which are known to be involved in the salicylic acid (SA) signaling pathway [42], were both significantly down-regulated in *exo70B1-1* and *exo70B1-2* compared to the wild type (Figure 4C,D).

In addition, we expressed the full-length coding sequence of *OsExo70B1* under the control of the native promoter in the *exo70B1-1* mutant. We then used the positive T_1_ complementary plants, which were identified by PCR with specific primers before use (Figure S3), for blast resistance analysis. After inoculating with the *M. oryzae* isolate Guy11, the complementary transgenic plants of *exo70B1-1* were similar to those in the wild type in the lesion area and fungal biomass analysis (Figure 4E,F), as well as the expression level of *OsPR5* and *OsPR10* (Figure 4G,H).

Taken together, the results above indicate that knocking out Exo70B1 decreased plant resistance to *M. oryzae* in rice.

### 2.4. Accumulation of H_2_O_2_ Is Lower and the Infection Progress Is Faster in exo70B1-1 Mutant Compared to the Wild Type

**Figure 5:**
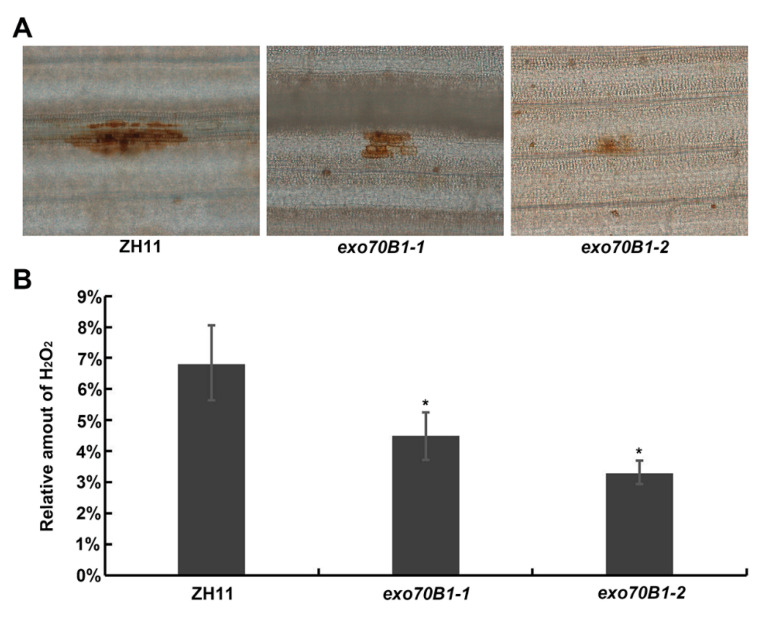
Accumulation of H_2_O_2_ is lower in *exo70B1-1* and *exo70B1-2* mutants compared to the wild type. (**A**) DAB (3,3′-diamino-bezidine) staining at infection sites of *exo70b1-1*, *exo70B1-2* and ZH11 plants 2 days post-inoculation (dpi). Darker staining indicates accumulation of H_2_O_2_. (**B**) Relative amount of H_2_O_2_ was calculated based on pixels taken with ImageJ using the following formula: H_2_O_2_ area per rectangle = pixel of H_2_O_2_ area per leaf/pixel of rectangle. Data are represented as the mean ± standard error of mean (SEM). Asterisks represent significant differences (* *p* < 0.05, Student’s *t*-test). Bar = 10 μm.

Accumulation of H_2_O_2_ is a common defense response to *M. oryzae* in rice [43,44,45]. To test whether H_2_O_2_ accumulation is different in exo70B1 mutants compared to the wild type, staining of the inoculated leaf cells with 3,3′-diamino-bezidine (DAB) for H_2_O_2_ revealed that the two allelic mutants *exo70B1-1* and *exo70B1-2* both produced much lower amounts of H_2_O_2_ in the inoculated leaf sheath cells than the wild type (Figure 5). This result suggests that decreased reactive oxygen species (ROS) burst may be part of the reason why *exo70b1-1* and *exo70b1-2* mutants display enhanced susceptibility to rice blast fungus.

**Figure 6:**
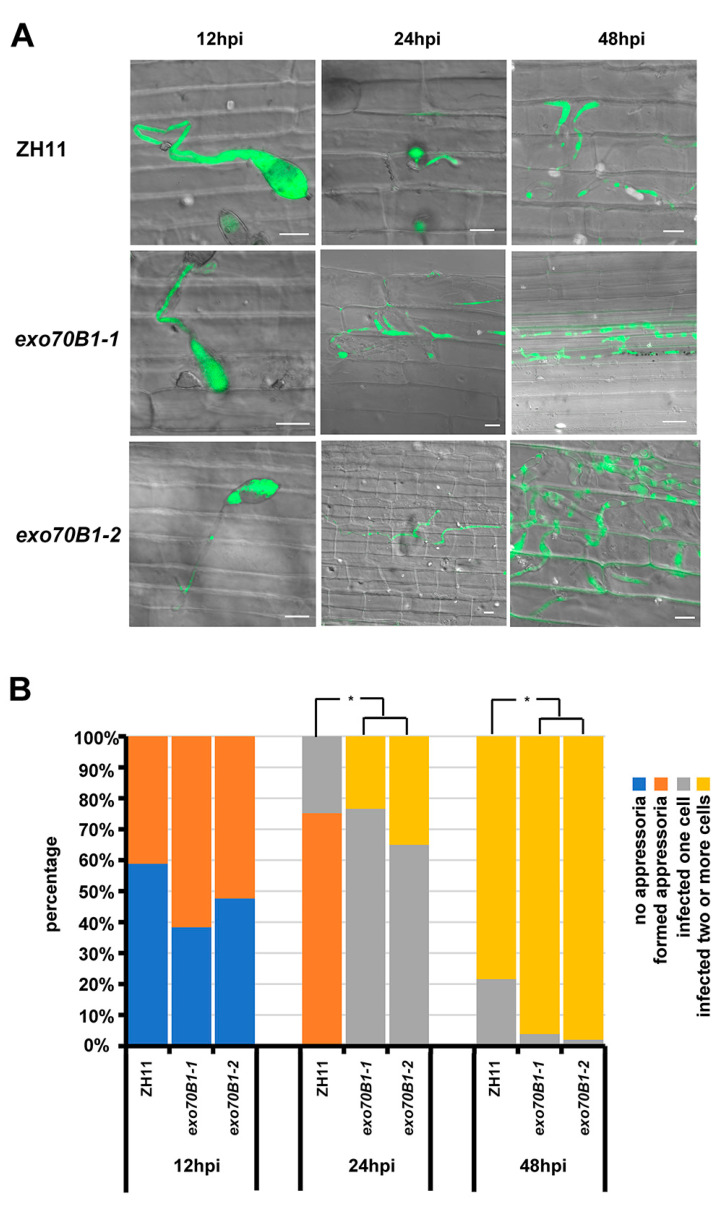
The infection progress of the enhanced green fluorescent protein (eGFP)-tagged *M. oryzae* isolate Zhong1 in *exo70B1-1*, *exo70B1-2* and ZH11 leaf sheath cells at different time periods after inoculation. (**A**) Representative laser scanning microscopy images of ZH11, *exo70B1-1* and *exo70B1-2* leaf sheath cells infected by the eGFP-tagged *M. oryzae* isolate Zhong1. Bar = 10 μm. (**B**) Distribution of fungal infection progression at 12, 24, and 48 hpi. At least 30 single-cell interaction sites were examined per replication. Each bar represents the mean of three replications. Asterisks represent significant differences (* *p* < 0.05, Student’s *t*-test).

To gain more insight into how *exo70B1-1* mutants are more susceptible to blast fungi compared to the wild type, the formation and expansion of invasive hyphae in leaf sheath cells were observed and quantified. Consistent with the decreased disease phenotypes, the infection progress occurred significantly earlier in *exo70B1-1* and *exo70B1-2* compared with the wild type (Figure 6). After leaf sheath injection with the eGFP-tagged *M. oryzae* strain Zhong1, we found that in ZH11, 41.1% of the spores of *M. oryzae* formed appressoria and 58.9% of the spores did not form appressoria at 12 hpi, while 61.6% of the spores in *exo70B1-1* and 56.3% in *exo70B1-2* formed appressoria and 38.4% in *exo70B1-1* and 43.7% in *exo70B1-2* did not form appressoria. At 24 hpi, only 24.8% of spores formed invasive hyphae, while the remaining 75.2% of appressoria did not invade cells in ZH11; however, in *exo70B1-1*, 76.6% of spores formed infective hyphae, and 23.4% of them expanded into neighbor cells. Similarly, 65.0% spores formed infective hyphae in *exo70B1-2* and 35.0% of them expanded into neighbor cells. Moreover, 78.3% of the infected hyphae in ZH11 had expanded to neighboring cells, while 96.1% of infective hyphae in *exo70B1-1* and 97.9% in *exo70B1-2* had expanded to more adjacent cells at 48 hpi. In conclusion, the infection progress is significantly faster in the *exo70B1-1* mutant compared to the wild type.

### 2.5. Knocking out OsExo70B1 Does Not Alter Plant Architecture or Grain Yield

**Figure 7:**
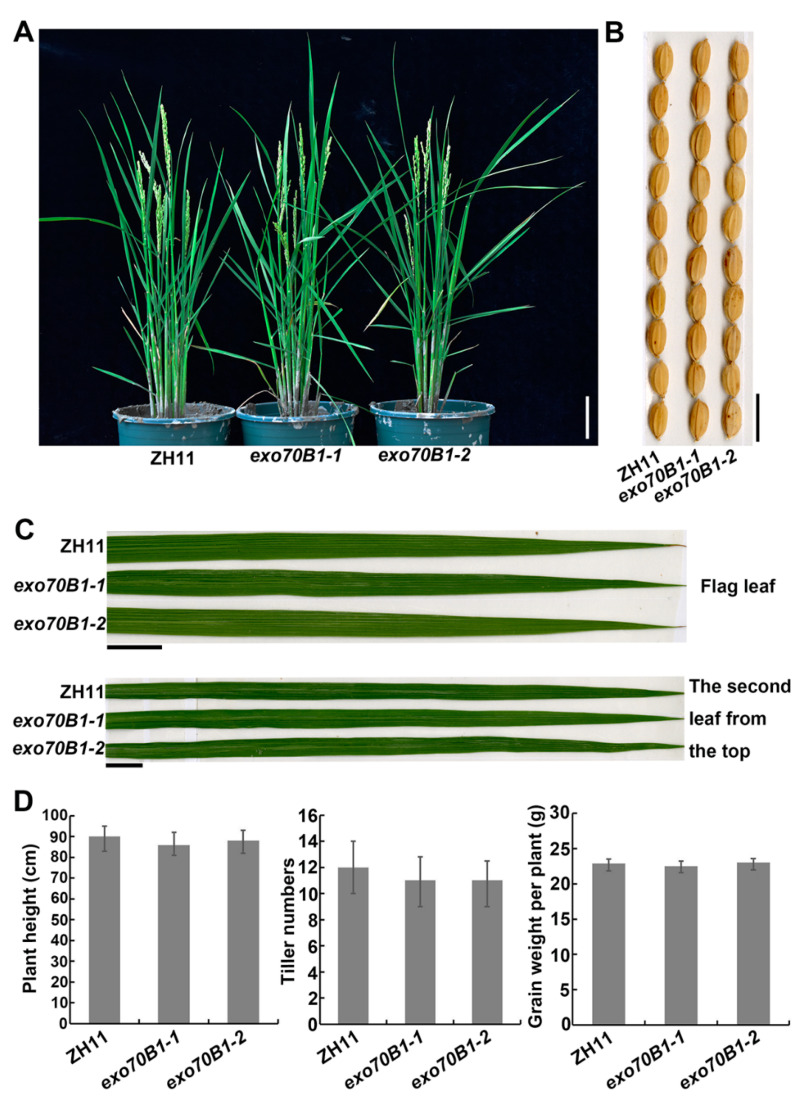
Growth and development phenotypes of the *exo70B1-1* and *exo70B1-2* mutants. (**A**) The plant architecture of ZH11, *exo70B1-1* and *exo70B1-2* at the heading stage. Bar = 15 cm. (**B**) Photograph of the seeds of ZH11, *exo70B1-1* and *exo70B1-2*. Bar = 1 cm. (**C**) Photograph of the flag leaf and top second leaf of the 4-week-old ZH11, *exo70B1-1* and *exo70B1-2*. Bar = 2 cm. (**D**) The plant height, tiller number and grain weight per plant of ZH11, *exo70B1-1* and *exo70B1-2*. Each bar represents the mean of twenty plants.

In *Arabidopsis*, it has been reported that *exo70B1-1* grew normally up to approximately 4 weeks after germination and then became smaller than the wild type and displayed HR-like cell death lesions [23,30,41]. Therefore, we also investigated the phenotypes of the rice *exo70B1* mutant in growth and development. As shown in Figure 7, *exo70B1-1* and *exo70B1-2* grew normally until the ripening phase (Figure 7A), and no cell death lesions appeared in *exo70B1-1* and *exo70B1-2* leaves (Figure 7C). Moreover, *exo70B1-1* showed no significant differences in plant height, tiller number, seed size and grain weight per plant compared to the wild type (Figure 7), indicating that knocking out Exo70B1 does not affect growth and development in rice.

### 2.6. OsExo70B1 Associates with OsCERK1

**Figure 8:**
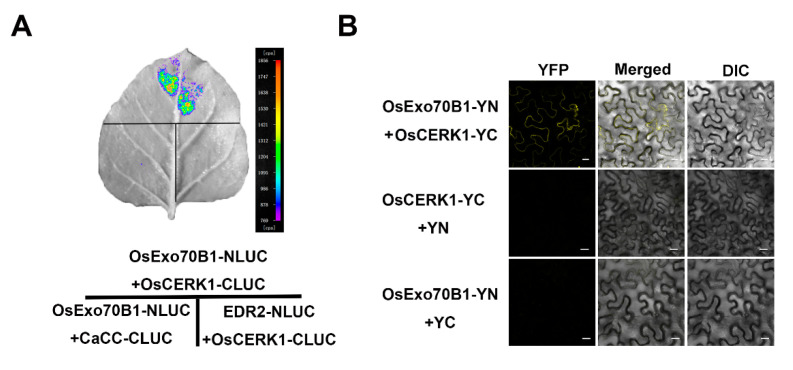
*OsExo70B1* associates with OsCERK1. (**A**) The interaction between *OsExo70B1* and OsCERK1 was examined with the split-luciferase complementation assay in *N. benthamiana*. Co-infiltrated of *OsExo70B1* fused with N-terminus of the Renilla luciferase fragment and OsCERK1 fused with the C-terminus of the Renilla luciferase fragment in *N. benthamiana* leaves by agrobacterium tumefaciens strain GV3101 mediated transformation. At the second day after injection, the infiltrated leaves were sprayed with 1 mM luciferin and the fluorescence signal was captured by a cooled charge coupled device (CCD) camera. The combinations of *OsExo70B1*-CaCC and OsCERK1-EDR2 were used as the controls. (**B**) The interaction between *OsExo70B1* and OsCERK1 was examined with the BiFC assay in *N. benthamiana*. *OsExo70B1* and OsCERK1 were fused to the N-terminus of the yellow fluorescent protein (YFP) fragment (YN) or the C-terminus of the YFP fragment (YC), respectively, and agrobacterium tumefaciens strain GV3101 containing the indicated construct pairs were co-infiltrated into *N. benthamiana* leaves. YFP fluorescence was detected by confocal microscopy. Bar = 20 μm.

It has been shown that the exocyst complex plays important roles in vesicle trafficking, specifically tethering and spatially targeting post-Golgi vesicles to the plasma membrane prior to vesicle fusion. Recently, Wang et al. showed that Arabidopsis Exo70B1 regulates the trafficking of FLS2, the receptor of bacterial flagellin, to the PM, thus mediating the immunity responses in *Arabidopsis* [46]. We speculate that *OsExo70B1* may also be responsible for the membrane location of some defense-related proteins in rice. As OsCERK1 is a well-characterized receptor-like kinase (RLK) and mediates the signal of chitin reception by coordinating with a lysin motif (LysM)-containing protein CEBiP [37], we first performed a split-luciferase complementation imaging (LCI) assay in *N. benthamiana*. As shown in Figure 8A, luminescent signals were detected when OsCERK1 was co-transformed with *OsExo70B1*, but not with the negative controls. Then, a bimolecular fluorescence complementation (BiFC) assay also showed that *OsExo70B1* can interact with OsCERK1 in the plasma membrane predominantly (Figure 8B). Taken together, the results above showed that *OsExo70B1* associated with OsCERK1 in BiFC and LCI assays.

## 3. Discussion

EXO70B1 is a component of the exocyst complex and belongs to the EXO70 protein subfamily. In this study, we characterized the expression pattern and function of *OsExo70B1* in rice, revealing its important roles in plant immune response in rice. *OsExo70B1* mainly expressed in leaves and its expression level can be induced by PAMPs and rice blast fungus (Figure 1 and Figure 3). Knocking out *OsExo70B1* resulted in decreased plant resistance to *M. oryzae*, as well as decreased H_2_O_2_ accumulation, downregulation of PR genes and more fungi growth (Figure 4 and Figure 5). However, we did not observe any defects associated with growth and development in *exo70B1* mutant (Figure 7), indicating that *OsExo70B1* mainly plays important roles in rice immunity regulation.

Several studies have shown the important roles of Exo70B1 in immune regulation in Arabidopsis [21,23,30,31,41]. However, there are some differences of Exo70B1 in rice and *Arabidopsis*. First, under standard short-day conditions, *Arabidopsis exo70B1* mutants grew normally up to 4 weeks of age. At this time point, *exo70B1* plants started to become smaller compared to the wild type, and displayed hypersensitive response-like cell death after 5 weeks [23,30,41]. However, the rice *exo70B1* mutant grew normally throughout its life cycle and no cell death appeared in its leaves (Figure 7). Second, *Arabidopsis exo70B1* loss-of-function mutants emerged activated defense responses upon infection and enhanced resistance to fungal, oomycete and bacterial pathogens [23]. By contrast, knocking out *OsExo70B1* compromises plant resistance in rice, and the rice *exo70B1* mutants displayed enhanced susceptibility to *M. oryzae*. Further study showed that enhanced disease resistance and cell death in *Arabidopsis exo70B1* mutant are dependent on TN2, a truncated TIR-NBS-LRR intracellular immune receptor. TN2 interacted with Exo70B1 directly, and *TN2* transcripts accumulate at much higher levels in the *exo70B1* mutant [23]. TN2 belongs to the TIR-NBS (TN) family, which has 21 members in *Arabidopsis* ecotype Col-0 [47], but TIR-NBS-LRR and TIR-NBS proteins are absent from cereal species, including rice [48], which may explain why no cell death occurred in rice *exo70B1* mutant. Thirdly, *Arabidopsis* Exo70B1 is reported to be involved in autophagy-related membrane traffic to the vacuole, and the *exo70B1* mutant displays ectopic hypersensitive reaction mediated by salicylic acid (SA) accumulation, as well as defects in autophagy-related phenotypes [30], but no cell death appeared in rice *exo70B1* mutants, and although we did not examine the autophagy-related phenotypes of rice *exo70B1* mutants, those mutants did not show growth defects, suggesting that there is no defect in autophagy-related pathways in rice *exo70b1* mutants. Therefore, *OsExo70B1* may not participate in autophagy-related membrane traffic in rice or some other proteins, such as *OsExo70B2* and *OsExo70B3*, function redundantly. In addition, Exo70B1 also participates in ABA mediated stomatal closure and light-induced stomatal opening in Arabidopsis [24,49]. It would be interesting to know whether *OsExo70B1* is also involved in stomatal opening and closure regulation in rice.

How Exo70B1 functions in plant immunity has been extensively studied in Arabidopsis. It is reported that RIN4 can recruit Exo70B1 to the plasma membrane and AvrRpt2 can release both RIN4 and Exo70B1 to the cytoplasm, indicating that the localization of Exo70B1 at PM may be required for plant immunity [39]. The plant U-box-type ubiquitin ligase 18 (PUB18) ubiquitinates and degrades Exo70B1 in ABA-mediated stomatal movement regulation [24]. Exo70B1 is also ubiquitinated by bacterial effector AvrPtoB after Pto DC3000 infects *Arabidopsis* [34]. Furthermore, the activated immune responses in the *Arabidopsis exo70B1* mutant require TN2 and CPK5 [23,31]. Most recently, Wang et al. (2020) found that both Exo70B1 and Exo70B2 are involved in the regulation of FLS2 accumulation at the PM. Moreover, Exo70B1 is also responsible for the membrane accumulation of BRI1 and CERK1, but not of RLK902-GFP, indicating that the exocyst complex subunits can regulate the trafficking of some defense-related proteins to the PM, thus mediating the immunity responses [46]. In this study, we detected the interaction between *OsExo70B1* and OsCERK1 in LCI and BiFC assays (Figure 8), suggested that *OsExo70B1* may also be involved in rice immunity by affecting the accumulation of some important PRRs, including OsCERK1, to the plasma membrane. However, the mechanisms of how *OsExo70B1* regulates immunity are still largely unknown. It would be interested to examine whether *OsExo70B1* contributes to the accumulation of OsCERK1 and other receptors of PAMPs at the PM.

Interestingly, there are only two *Exo70B* family genes in *Arabidopsis*, *Exo70B1* and *Exo70B2*, while there are three *Exo70B* family genes in rice [17]. In *Arabidopsis*, Exo70B2 also has been reported to function in immunity regulation [21,29]. Exo70B2 also contributes to the accumulation of FLS2 at the PM [46]. However, the FLS2 accumulation level at the PM in the *exo70B1-3exo70B2-1* double mutant was not further reduced relative to that in *exo70B1-3* and *exo70B2-1* single mutants and Exo70B1 can form a heterodimer with Exo70B2, indicating that Exo70B1 and Exo70B2 cooperatively function in trafficking FLS2 to the PM [46]. In rice, the role of three Exo70B family genes is not studied, and their functions are still unknown. Here, we showed that *OsExo70B1* positively regulates rice immunity. Though whether *OsExo70B2* and *OsExo70B3* have a similar function, and whether the three *Exo70B* genes function redundantly, still require further studies. In addition, whether the HR-like cell death not observed in the rice *exo70B1* mutant is due to the redundancy of rice Exo70Bs remains to be determined. It would be interesting to examine the triple mutant of *OsExo70B1*, *OsExo70B2* and *OsExo70B3*, and the characterization of the phenotypes of the triple mutants will help us to understand the functions of *Exo70B* genes in rice.

Our data collectively indicate the positive regulation roles of *OsExo70B1* in rice immunity, and they may function by mediating the trafficking and enrichment of OsCERK1 on the membrane. Then OsCERK1 mediated immune responses, including ROS burst and PTI-related defense genes expression, were activated, which ultimately lead to enhanced disease resistance in rice.

## 4. Materials and Methods

### 4.1. Plant Materials and Growth Conditions

Rice and *Nicotiana benthamiana* (*N. benthamiana*) plants were used in this study. The *exo70B1-1* and *exo70B1-2* mutants were constructed with the CRISPR/Cas9 system in Zhonghua 11 (ZH11) [50], and the sgRNA for *exo70B1-1* is 5′-GATCTCGCAGTTCGTGACGA-3′ and for *exo70B1-2* is 5′-GGGACAAACTCTACGCCACG-3′. All rice plants used in this study were grown in paddy fields of the Fujian Agriculture and Forestry University in Fuzhou, Fujian province, paddy fields of the Institute of Crop Science in Sanya (China), or a greenhouse at 25–28 °C with a 16 h:8 h, light:dark photoperiod. *N. benthamiana* plants were growth in a greenhouse at 22 °C with 12 h:12 h, light:dark photoperiod.

### 4.2. Subcell Localization Analysis

The full-length cDNA sequence of *OsExo70B1* was amplified and inserted between EcoRI and SmaI sites of the pCMABIA2300-35S-eGFP vector with an in-fusion PCR cloning kit, thus producing the 35S-*OsExo70B1*-eGFP construct. Then, the constructs were transformed into agrobacterium strain GV3101 and injected into *N. benthamiana* leaves. A GFP signal was observed and photographed with a Zeiss 880 confocal microscope at 3 days after injection.

### 4.3. Construction of Complementary Transgenic Plant of exo70B1-1

To produce the *OsExo70B1pro:OsExo70B1-GFP* construct, 1511 bp of the *OsExo70B1* promoter sequence, together with the full length of the *OsExo70B1* cDNA sequence without the stop codon, was amplified and inserted between EcoRI and KpnI, and KpnI and SpeI sites of the pCAMBIA2300 vector with in-fusion PCR cloning kit, respectively. Then, the construct was introduced into *Agrobacterium tumefaciens* strain *EHA105* and then transferred into the *exo70B1-1* mutant as described previously [51].

### 4.4. Gene Expression Analysis

Total RNA was isolated using TRIzol (Invitrogen) according to the manufacturer’s instructions, and then was synthetized into cDNA with an RT reagent Kit (code number is RR047A; Takara, Dalian, China) for qRT-PCR and 1st Strand cDNA Synthesis Kit for RT-PCR (code number is 6110A; Takara, Dalian, China). All qRT–PCR assays were performed with the Premix Ex TaqKit (code number is RR420A; TaKaRa, Dalian, China) in a CFX Connect Real-time PCR System (BIO-RAD, Hercules, CA, USA). RT-PCR assays were performed with the Ex Taq DNA Polymerase (code number is RR001A; TaKaRa, Dalian, China) in a T100 PCR System (BIO-RAD, Hercules, CA, USA). All of the primers used here are listed in Supplementary Table S1. qRT-PCR analysis was performed as previously described [52] with three independent biological replicates.

### 4.5. Chitin and flg22 Treatments

Chitin and flg22 treatment assays were performed as described previously [53]. When the wild-type ZH11 seedings grew to four weeks, the leaf segments that were 2–3 cm in length were collected and balanced in ddH_2_O for 12 h, and then they were treated with 1 µM flg22 (synthesized by Sangon Biotech Co., Ltd., Shanghai, China) or 20 µg/mL chitin (Sigma, St. Louis, MO, USA). The samples were collected at 6 h and 12 h after treatment for further analysis.

### 4.6. Rice Blast Fungus Incubation and Rice Sheath Penetration Assay

Blast fungus inoculation and a rice sheath penetration assay were performed as described previously with minor modification [54,55]. Briefly, two-week-old seedlings were sprayed with a spore suspension (1 ×10^5^ spores/mL) of the *M. oryzae* isolate Guy11. The inoculated plants were placed into dark and humid containers for 24 h. The plants were then transferred to a humidity growth chamber and grown under a 12 h:12 h, light:dark photoperiod. The disease lesions were examined and scored at 3 or 4 days after inoculation. The fungal biomass assay was performed as described previously [42,45,53]. Total DNA was extracted from ten diseased leaves of both ZH11 and *exo70B1* mutants, respectively, and then the fungal biomass was calculated by comparing the total DNA of *M. oryzae Pot2*, an inverted repeat transposon and represents one of the major repetitive DNAs in *M. oryzae* [56], to the *Ubiquitin* DNA of rice. Moreover, for rice sheath inoculation assays, spores of Zhong1 tagged with GFP were diluted to a concentration of 1 × 10 ^5^ spores/mL and injected into the inner leaf sheath of 3-week-old ZH11 plants. Then the inoculated leaf sheaths were incubated in a dark and humid container at 28 ℃. At 12 h, 24 h and 48 h after infection, the fungal growth in rice leaf sheath cells was observed under a fluorescence microscope.

### 4.7. DAB Staining

DAB staining was performed as described before [54]. Briefly, the leaf sheaths were immersed in 1 mg/mL DAB (pH = 3.8) at the indicated time after inoculation with *M. oryzae*. After vacuum infiltration for 30 min, the samples were incubated at room temperature for 12 h in the dark. When the brown spots appeared clearly, samples were bleached by boiling in ethanol:lactic acid:glycerol (1:1:1, v/v/v) for 15 min. Images were captured using a microscope with a CCD camera (Olympus BX51, Tokyo, Japan).

### 4.8. Split-Luciferase Complementation Assay

For the split-luciferase complementation assay, the full-length CDSs of *OsExo70B1* and *OsCERK1* were amplified with specific primers (Supplementary Table S1) and then cloned into both the KpnI and SalI sites of NLuc and CLuc vectors, respectively [57], producing the 35Spro-*OsExo70B1*-NLUC and 35Spro-OsCERK1-CLUC constructs. Pairs of *Agrobacterium tumefaciens* GV3101 strains containing the desired plasmid combinations were co-transformed into 4-week-old *N. benthamiana* leaves and incubated in the green room for 72 h. The assay was then performed as previously described [58]. The *N. benthamiana* leaves were sprayed with 1 mM luciferin and placed in darkness for 5 min. Subsequently, LUC images were captured with a low-light cooled CCD imaging apparatus.

### 4.9. Bimolecular Fluorescence Complementation (BiFC) Assay

The BiFC assay was performed as previously described [57]. Briefly, the coding sequences of *OsExo70B1* and *OsCERK1* were cloned into pDONR207 ENTRY vector first, and then into the pSPYNE and pSPYCE vectors respectively, in-frame with the N- and C-terminus of the yellow fluorescent protein (YFP) with the gateway cloning system, thus producing 35Spro-*OsExo70B1*-YN and 35Spro-OsCERK1-YC constructs. Then, agrobacterium strain GV3101 containing the respective plasmids was injected into the leaves of 4-week-old *N. benthamiana* plants. YFP signal was detected with a Zeiss 880 confocal microscope on the third day after injection.

## 5. Conclusions

In this study, we characterized the function of *OsExo70B1*, the subunit of exocyst in rice, revealing its important roles in plant immune response. Knocking out *OsExo70B1* resulted in enhanced susceptibility to *M. oryzae*, decreased H_2_O_2_ accumulation, and downregulation of PR genes. However, we did not observe any defects associated with growth and development in *exo70B1* mutant, which is in contrast to the autoimmune phenotypes in *Arabidopsis exo70B1* mutant. Moreover, we find *OsExo70B1* interacts with OsCERK1, an essential component for chitin reception in rice. Our findings reveal the positive roles of *OsExo70B1* in rice immunity regulation, as well as the functional differentiation of Exo70B1 in *Arabidopsis* and rice.

## Supplementary Materials

Supplementary materials can be found at https://www.mdpi.com/1422-0067/21/19/7049/s1

## Gene Accession Numbers

Sequence data from this article can be found in Rice Genome Annotation Project databases under the following accession numbers:	
*OsExo70B1*	(LOC_Os01g61180)
*OsCERK1*	(LOC_Os08g425880)
*Ubiquitin*	(LOC_Os03g13170)
*Actin*	(LOC_Os03g50885)
*PR5*	(LOC_Os03g46070)
*PR10*	(LOC_Os12g36880)
*Pot2*	(EMBL accession Z33638)
