# Supplementary figures and images for: OsExo70B1 Positively Regulates Disease Resistance to Magnaporthe oryzae in Rice
Source: Int J Mol Sci. 2020 Sep 25;21(19):7049. doi: 10.3390/ijms21197049 (PMC7582735; doi:10.3390/ijms21197049)

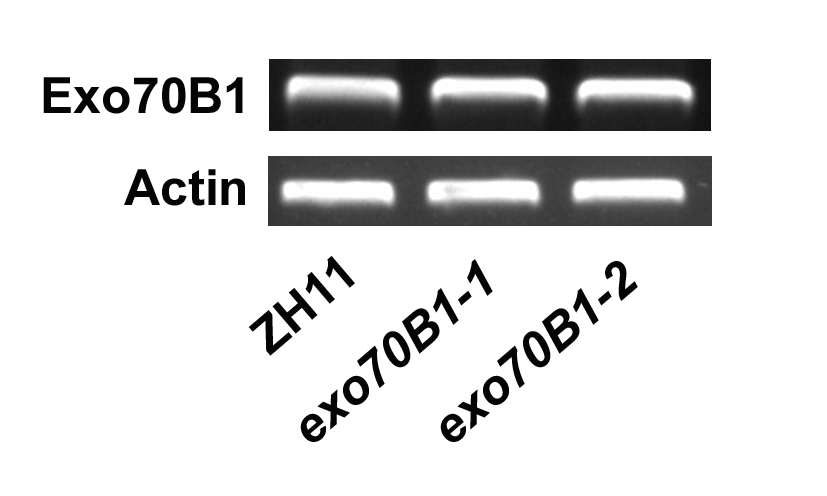

Supplement: Supplementary file 1 [file ijms-21-07049-s001.zip › Figure S2.tif]

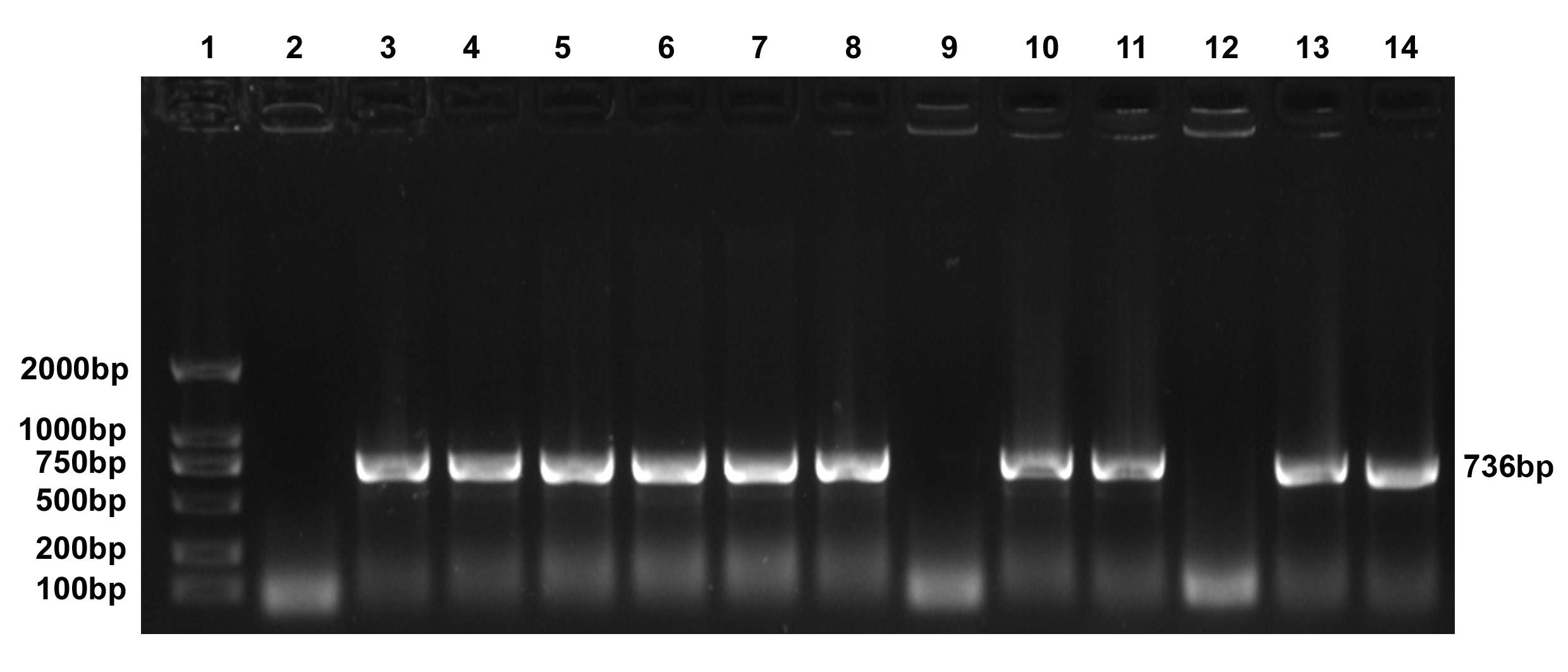

Supplement: Supplementary file 1 [file ijms-21-07049-s001.zip › Figure S3.tif]

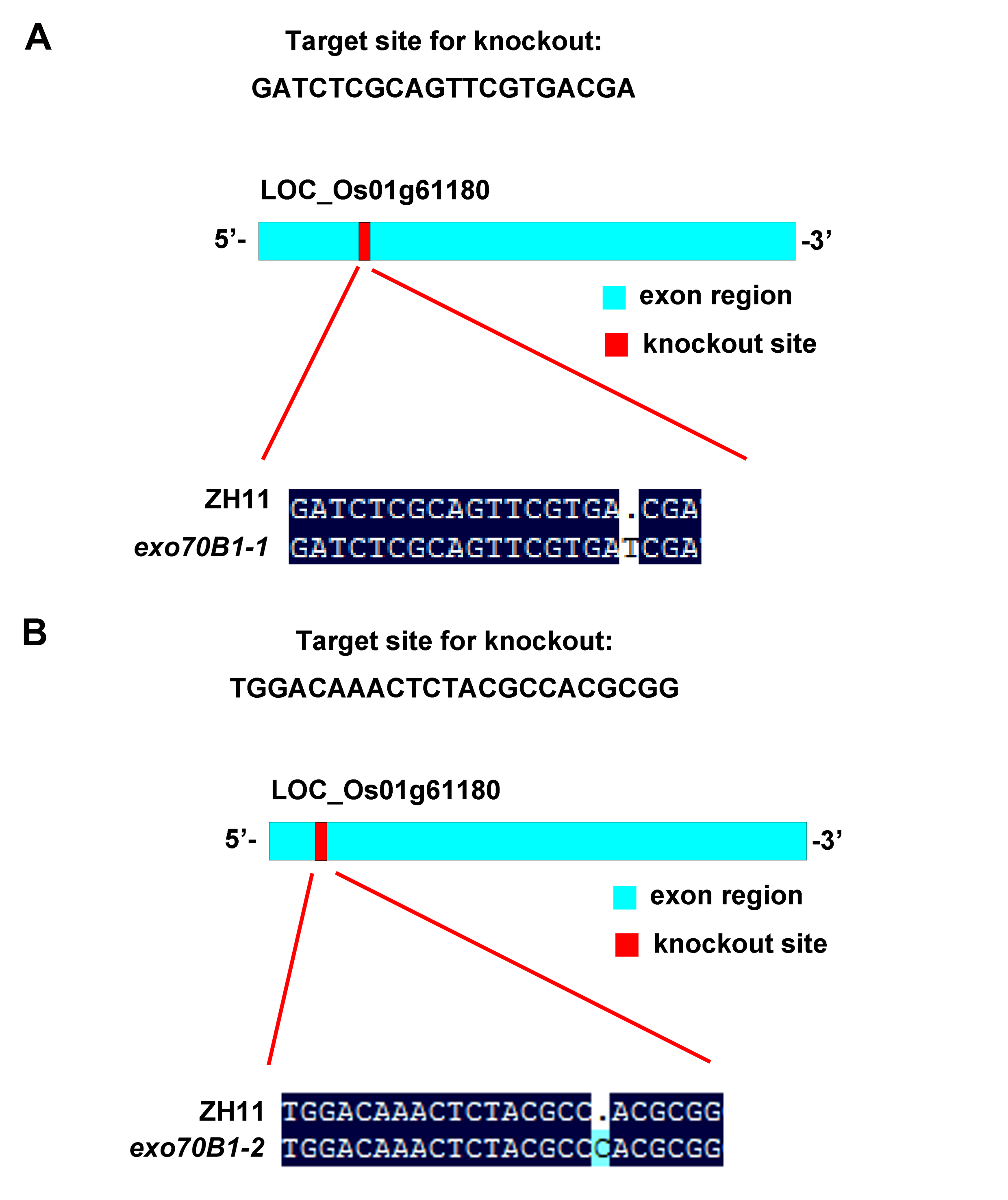

Supplement: Supplementary file 1 [file ijms-21-07049-s001.zip › FIgure S1.tif]
