# Supplementary material for: OsExo70B1 Positively Regulates Disease Resistance to Magnaporthe oryzae in Rice
Source: Int J Mol Sci. 2020 Sep 25;21(19):7049. doi: 10.3390/ijms21197049 (PMC7582735; doi:10.3390/ijms21197049)
Supplement: Supplementary file 1 [file ijms-21-07049-s001.zip › Supplementary Materials revised.doc]

**Supplementary Materials**

**Figure S1.** Determination of *exo70B1-1* and *exo70B1-2* knockout transgenic lines

The target sites of the Os*Exo70B1* gene were knocked out using the CRISPR/Cas9 system, and the gene knockout plants were verified by PCR sequencing.

(**A**) The mutation site of *exo70B1-1* indicates that there is a T base insertion at the 245 position, which results in a code-shift mutation from Pro_84._

(**B**) The mutation site of *exo70B1-2* indicates that there is a C base insertion at the 145 position, which results in a code-shift mutation from His_49._

**Figure S2.** RT-PCR analysis of the expression of *OsExo70B1* in the two *exo70B1* mutants

Total RNA was isolated from the leaves of 4-week-old seedlings of ZH11, *exo70B1-1* and *exo70B1-2* respectively and synthetized into cDNA. And then the expression level of Os*Exo70B1*, set the *Actin* as a control, in ZH11 and the two mutants were analyzed by RT-PCR. It is showed that *OsExo70B1* still expressed in two mutants.

**Figure S3.** Identification of positive T_1_ complementary plants of the two complementary plants (CP) lines by PCR with specific primers

When the T_1_ complementary plants grew to two weeks old. The leaf segments with 2-3 cm in length from the top second leaf were collected and broken by tissue grinder. Then broken leaves were added about 50 μL TE buffer and used as PCR template. The positive complementary plants can be amplified a 736 bp DNA fragment, but the negative complementary plants could not.

Lane 1, Marker; Lane 2, ZH11; Lane 3 to 14, the T_1_ complementary plants.

**Table S1.** List of primers used in this work.
